# Clinical Characteristics and Neonatal Outcomes of Pregnant Patients With COVID-19: A Systematic Review

**DOI:** 10.3389/fmed.2020.573468

**Published:** 2020-12-03

**Authors:** Md. Mohaimenul Islam, Tahmina Nasrin Poly, Bruno Andreas Walther, Hsuan Chia Yang, Cheng-Wei Wang, Wen-Shyang Hsieh, Suleman Atique, Hosna Salmani, Belal Alsinglawi, Ming Ching Lin, Wen Shan Jian, Yu-Chuan Jack Li

**Affiliations:** ^1^College of Medical Science and Technology, Graduate Institute of Biomedical Informatics, Taipei Medical University, Taipei, Taiwan; ^2^International Center for Health Information Technology (ICHIT), Taipei Medical University, Taipei, Taiwan; ^3^Research Center of Big Data and Meta-Analysis, Wan Fang Hospital, Taipei Medical University, Taipei, Taiwan; ^4^Alfred-Wegener-Institut Helmholtz-Zentrum für Polar- und Meeresforschung, Bremerhaven, Germany; ^5^Division of Reproduction Medicine, Department of Obstetrics and Gynecology, Taipei Medical University Hospital, Taipei, Taiwan; ^6^Department of Medical Laboratory, Shuang Ho Hospital, Taipei Medical University, New Taipei City, Taiwan; ^7^Department of Medical Laboratory Science and Biotechnology, Fooyin University, Kaohsiung City, Taiwan; ^8^College of Public Health and Health Informatics, University of Ha'il, Ha'il, Saudi Arabia; ^9^School of Health Management and Information Sciences, Iran University of Medical Sciences, Tehran, Iran; ^10^School of Computing, Engineering, and Mathematics, Western Sydney University, Penrith, NSW, Australia; ^11^Department of Neurosurgery, Shuang Ho Hospital, Taipei Medical University, Taipei, Taiwan; ^12^Professional Master Program in Artificial Intelligence in Medicine, Taipei Medical University, Taipei, Taiwan; ^13^Research Center for Artificial Intelligence in Medicine, Taipei Medical University, Taipei, Taiwan; ^14^Taipei Neuroscience Institute, Taipei Medical University, Taipei, Taiwan; ^15^School of Health Care Administration, Taipei Medical University, Taipei, Taiwan; ^16^Department of Dermatology, Wan Fang Hospital, Taipei, Taiwan; ^17^Taipei Medical University Research Center of Cancer Translational Medicine, Taipei Medical University, Taipei, Taiwan

**Keywords:** coronavirus, COVID-19, pregnant women, SARS–CoV-2, CT-scan

## Abstract

**Background and Objective:** Coronavirus disease 2019 (COVID-19) characterized by severe acute respiratory syndrome coronavirus 2 (SARS-CoV-2) has created serious concerns about its potential adverse effects. There are limited data on clinical, radiological, and neonatal outcomes of pregnant women with COVID-19 pneumonia. This study aimed to assess clinical manifestations and neonatal outcomes of pregnant women with COVID-19.

**Methods:** We conducted a systematic article search of PubMed, EMBASE, Scopus, Google Scholar, and Web of Science for studies that discussed pregnant patients with confirmed COVID-19 between January 1, 2020, and April 20, 2020, with no restriction on language. Articles were independently evaluated by two expert authors. We included all retrospective studies that reported the clinical features and outcomes of pregnant patients with COVID-19.

**Results:** Forty-seven articles were assessed for eligibility; 13 articles met the inclusion criteria for the systematic review. Data is reported for 235 pregnant women with COVID-19. The age range of patients was 25–40 years, and the gestational age ranged from 8 to 40 weeks plus 6 days. Clinical characteristics were fever [138/235 (58.72%)], cough [111/235 (47.23%)], and sore throat [21/235 (8.93%)]. One hundred fifty six out of 235 (66.38%) pregnant women had cesarean section, and 79 (33.62%) had a vaginal delivery. All the patients showed lung abnormalities in CT scan images, and none of the patients died. Neutrophil cell count, C-reactive protein (CRP) concentration, ALT, and AST were increased but lymphocyte count and albumin levels were decreased. Amniotic fluid, neonatal throat swab, and breastmilk samples were taken to test for SARS-CoV-2 but all found negativ results. Recent published evidence showed the possibility of vertical transmission up to 30%, and neonatal death up to 2.5%. Pre-eclampsia, fetal distress, PROM, pre-mature delivery were the major complications of pregnant women with COVID-19.

**Conclusions:** Our study findings show that the clinical, laboratory and radiological characteristics of pregnant women with COVID-19 were similar to those of the general populations. The possibility of vertical transmission cannot be ignored but C-section should not be routinely recommended anymore according to latest evidences and, in any case, decisions should be taken after proper discussion with the family. Future studies are needed to confirm or refute these findings with a larger number of sample sizes and a long-term follow-up period.

## Introduction

Coronavirus disease 2019 (COVID-19), a global health emergency, poses a serious public health threat (1). Countries are implementing public health measures such as effective and efficient preventive and control strategies (lock-down, social distancing, strict travel restriction, and shut down of academic institutions) to combat the ongoing coronavirus spread (2). Currently, there are no vaccines or direct-acting antiviral medications available for the treatment of SARS-CoV-2 infection (3). The concern has also been raised because the numbers of pregnant women with confirmed COVID-19 have been increasing, and viral pneumonia is considered as one of the main risk factors of severe illness, higher morbidity, and mortality among pregnant women (4). Previously, both severe acute respiratory syndrome coronavirus (SARS-CoV) and Middle East respiratory syndrome coronavirus (MERS-CoV) created severe complications in pregnant women (5, 6), and the mortality rate of SARS-CoV infection was 25% in pregnant women (4).

Several studies reported that maternal pneumonia is responsible for unfavorable obstetrical outcomes such as the pre-mature rupture of membranes (PROM), intrauterine fetal demise (IUFD), intrauterine growth restriction (IUGR), and neonatal death (6, 7). However, limited data exists for this population with COVID-19; it is not even well-established whether COVID-19 could transmit vertically and increase the risk to the fetus and neonate. We, therefore, aimed to summarize the epidemiological, clinical, laboratory, and radiological manifestations, and neonatal outcomes of pregnant women with COVID-19. This study highlighted the following research questions: (a) What are the clinical, laboratory, and radiological outcomes for the patients with COVID-19? (b) Does COVID-19 vertically transmit from mother to children? (c) Is COVID-19 an indication of cesarean section delivery for pregnant women?, and d) Does COVID-19 create severe maternal and neonatal complications among pregnant women?

## Methods

### Databases and Search Strategy

We systematically searched for published articles presenting data for pregnant patients with confirmed COVID-19 between January 1, 2020, and April 20, 2020, with no restriction on language. We searched the electronic databases such as PubMed, EMBASE, Scopus, and Google Scholar using the following search terms: “pregnant women or pregnant patients” and “COVID-19” or “Coronavirus disease-19” or “SARS-CoV-2.” One author initially conducted the relevant article search, which was later on verified by another expert author. We further scrutinized the reference lists from retrieved articles in order to find more potential articles.

### Eligibility Criteria

Eligibility was restricted to articles that examined the clinical characteristics like signs and symptoms, and laboratory findings, radiological features, and neonatal outcomes of pregnant women. We excluded studies if they did not invest in the neonatal outcome of pregnant patients with COVID-19. As for the study design, retrospective studies including case-control, letter to the editor, case study without any limitation of participants were eligible for inclusion, while we excluded studies that were only published in the form of abstracts or reviews.

### Study Selection

Our two experts (MMI, TNP) of systematic review and meta-analysis independently reviewed the titles and abstracts of the retrieved articles. They selected the appropriate articles based on the pre-specified selection criteria. Disagreements during this stage were settled by further discussion with the main supervisor (YCL). They also developed a data collection form to extract relevant information from selected articles. Furthermore, they checked retrieved articles duplication by comparing authors' names, publication date, journal names, study design, and participant's sizes.

### Data Extraction

Primary outcome measures were clinical features and neonatal outcomes of pregnant patients with SARS-CoV-2. Therefore, we collected epidemiological data, clinical signs and symptoms such as fever, cough, fatigue and diarrhea, laboratory results, chest radiology and CT findings, delivery methods (cesarean section or natural), neonates' outcomes such as birth weight, clinical symptoms, and Apgar score. The author's name, publication date, location, and the number of pregnant women and neonates were also collected.

### Statistical Analysis

Statistical analyses were conducted with Excel. We showed continuous variables directly as a range and categorical variables as a number (%).

## Results

The article search of the electronic databases extracted 237 articles. We excluded 190 articles after reviewing their titles and abstracts because they did not fulfill the selection criteria. A total of 47 articles went for full-text assessment, and we further checked their bibliography in order to find additional articles to include; however, no additional articles were found. We further excluded 34 studies because 23 studies did not meet in our inclusion criteria, 7 studies did not provide appropriate characteristics of pregnant women, 3 studies were reviews, and 1 study was a duplicate. Finally, 13 studies were included in our systematic review (8–20). The flow chart of our search strategy is illustrated in Supplementary Figure 1.

Twelve studies reported the gestational age of the pregnancy, and more than 95 percent of pregnant women were in their third trimester. There were 156/235 (66.38%) pregnant women that had a C-section delivery, and the patients' age ranged from 25 to 40 years. The most common pregnancy complications were gestational hypertension, pre-eclampsia, and PROM. However, some pregnant women experienced anemia, GDM, uterine scarring, or hypothyroidism. One hundred thirty eight out of 235 patients (58.72%) had a fever on admission, and 43 patients (18.30%) had a post-partum fever. Other symptoms were also observed in pregnant women. One hundred eleven patients had a cough, 21 patients had a sore throat, and 20 patients had fatigue. Furthermore, myalgia, malaise, rigor, sputum coryza, and diarrhea were also seen in pregnant women with COVID-19 (Table 1).

**Table 1:** Clinical features of 235 mothers with COVID-19 infection.

	**Chen**	**Cao**	**Chen**	**Wang**	**Yu**	**Wu$**	**Li**	Chen^*^	**Liu**	**Zhu**	**Lee**	**Yang**	**Yang**
**Clinical characteristics**													
**Age**													
Avg ± SD.	29.88 ± 4.83	30.3 ± 1.82	28.8 ± 2.28	28	32.17 ± 2.11	29.21 ± 4.16	30.9 ± 3.2	–	32 ± 5	30.7 ± 3.12	28	–	30.2 ± 2.3
Range	26–40	29–35	25–31	–	29–34	21–37	26–37	28–34	23–40	25–35	–	–	–
Epidemiologic history	9 (100%)	10 (100%)	–	1 (100%)	7 (100%)	23 (100%)		–	–		1 (100%)	–	–
**Complications**													
Influenza	1 (11.11%)	–	–	–	–	–	–	–	–	–	–	–	–
Gestational hypertension	1 (11.11%)	–	–	–	–	4 (17.39%)	3 (18.75%)	–	–	–	–	2 (28.57%)	–
Gestational diabetics	–	–	2 (40%)	–	–	–	3 (18.75%)	–	–	–	–	–	–
Pre-eclampsia	1 (11.11%)	3 (30%)	1 (20%)	–	–	–	1 (6.25%)	–	–	–	–	2 (28.57%)	–
Fetal distress	2 (22.22%)	2 (20%)	–	–	–	–	–	–	–	–	–	–	–
PROM	2 (22.22%)	4 (40%)	–	–	–	–	1 (6.25%)	–	–	–	–	–	–
Pre-mature	–	3 (30%)	–	1 (100%)	–	–	1 (6.25%)	–	–	3 (30%)	–	–	–
Uterine scarring	–	–	–	–	3 (42.85%)	–	–		–	6 (60%)	–	–	–
Placental abruption	–	1 (10%)	–	–		–	–	–	–	1 (10%)	–	–	–
Hypothyroidism	–	1 (10%)	–	–	–	2 (8.70%)	2 (12.5%)	–	–	–	–	–	–
Anemia	–	1 (10%)	–	–	–	–	–	–	–	–	–	–	–
GDM	–	1 (10%)	–	–	–	–	–	–	–	–	–	–	–
Twins	–	1 (10%)	–	–	–	–	–	–	–	–	–	–	–
None	2 (22.22%)	–	2 (20%)	–	4 (57.15%)	–	–	–	–	–	–	–	–
**Signs and symptoms**													
Fever on admission	7 (78%)	2 (20%)	–	–	7 (85.71%)	4 (17.4%)	4 (25.0%)	84 (75%)	13 (86.66%)	9 (90%)	1 (100%)	5 (71.42%)	2(15.4%)
Post-partum fever	6 (67%)	5 (50%)	5 (100%)	–	7 (85.71%)	–	8 (50.0%)	–	1 (6.66%)	3 (30%)	–	–	–
Myalgia	3 (33%)	–	–	–	–	–	–	–	3 (20%)	–	–	–	–
Malaise	2 (22%)	–	–	–	–	–	–	–	–	–	–	–	–
Rigor	–	–	–	–	–	–	–	–	–	–	–	–	–
Cough	4 (44%)	1 (10%)	1 (20%)	–	1 (14.28%)	6 (26. 1%)	–	82 (73%)	9 (60%)	5 (50%)	1 (100%)	1 (14.28%)	–
Sputum	–	–	1 (20%)	–	–	–	–	–	–	–	–	–	–
Coryza	–	–	1 (20%)		–	–	–	–	–	–	–	–	–
Dyspnea	1 (11%)	–	–	–	1 (14.28%)	–	–	8 (7%)	1 (6.66%)	–	–	–	–
Sore throat	2 (22%)	–	–	–	–	–	–	–	1 (6.66%)	1 (10%)	1 (100%)	–	–
Diarrhea	1 (11%)	–	–		1 (14.28%)	–	–	8 (7%)	1 (6.66%)	1 (10%)	–	1 (14.28%)	–
Chest pain	–	1 (10%)	–	–	–	–	–	20 (18%)	–	–	–	–	–
Fatigue	–	1 (10%)	–	–	–	–	–	19 (17%)	4 (26.66%)	–	–	–	–
**Delivery methods**													
C-section	9 (100%)	8 (80%)	2 (40%)	1 (100%)	7 (100%)	18 (90%)	14 (87.5%)	63 (93%)^**^	10 (90.90%)	7 (70%)	1 (100%)	7 (100%)	–
Vaginal birth	0	2 (2%)	3 (60%)	–	–	2 (10%)	2 (12.5%)	5 (7%)^**^	1 (9.90%)	3 (30%)	–	–	–
**Indication for C-section**													
Elevated ALT or AST	1 (11.11%)	0	–	–	–	–	–	–	–	–	–	–	–
Mature	1 (11.11%)	0	–	–	–	–	–	–	–	–	–	–	–
History of C-section	1 (11.11%)	2 (20%)	–	–	–	–	–	–	–	–	–	–	–
Pre-eclampsia	1 (11.11%)	1 (10%)	–	–	–	–	–	–	–	–	–	2 (28.57%)	–
Fetal distress	2 (22.22%)	3 (30%)	–	–	–	–	3 (18.75%)	–	–	–	–	–	–
History of stillbirth	1 (11.11%)	0	–	–	–	–	–	–	–	–	–	–	–
PROM	2 (22.22%)	2 (20%)	–	–	–	–	2 (12.5%)	–	–	4 (40%)	–	–	–
Cephalopelvic disproportion	–	–	–	–	–	–	–	–	–	–	1 (100%)	–	–
Twin	0	1 (10%)	–	–	–	–	2 (12.5%)	–	–	–	–	–	–
Concerned COVID−19				1 (100%)	–	20 (100%)	–	38 (61%)	–	–	–	–	–
None	0	2 (20%)	–	–	–	–	–	–	–	–	–	–	–

* six patients were asymptomatic;

** *delivered 68 out of 118; $, Twenty patients delivered*.

Five studies reported the white blood cell count (WBC); however, eight patients reported an elevated result. Data showed that 58 pregnant women with COVID-19 pneumonia had lymphopenia (<1·0 × 10^9^ cells per L). Sixty-three patients had elevated concentrations of CRP (>4 mg/L). Five had increased concentrations of alanine aminotransferase (ALT), 4 had increased concentration of aspartate aminotransferase (AST), and five had a higher level of alkaline phosphatase level (APL) (Table 2). Moreover, 6 patients had increased lactate dehydrogenase, and 46 patients experienced increased D-dimer concentration (>0.5 ug/L). All patients were tested positive by the confirmatory test (SARS-CoV-2 quantitative RT-PCR).

**Table 2:** Laboratory characteristics of 235 pregnant women with COVID−19.

	**Normal range**	**Chen**	**Cao**	**Chen**	**Wang**	**Yu**	**Wu $**	**Li**	Chen^*^	**Liu**	**Zhu**	**Lee**	**Yang**	**Yang**
White blood cell count (× 10^9^ cells per L)	3.5–9.5	7.79 ± 1.98 (2 patients, 22.22%)↑	8.93 ± 1.70 (3 patients, 30%)↑	11.22 ± 3.61 (3 patients, 60%)↑	N/A	N/A	N/A	8.6 ± 1.8	N/A	N/A	N/A	N/A	N/A	8.9 ± 1.5
Lymphocyte count (× 10^9^ cells per L)	1.1–3.2	1.18 ± 0.75 (5 patients, 55.56%)↓	1.13 ± 0.63 (6 patients, 60%)↓	0.92 ± 0.11 (4 patients, 80%)↓	0.86	0.90 ± 0.17^*^	0.77 ± 0.01	1.5 ± 0.4 (2 patients, 12.5%)↓	N/A	(12 patients, 80.0%)↓	N/A	N/A	N/A	1.4 ± 0.4
Neutrophil cell count (NEUT), × 109/L	1.8–6.3	N/A	N/A	9.53 ± 3.76 (4 patients, 80%)↑	9.14↑	11.43 ± 0.25↑	16.03 ± 0.30↑	6.6 ± 1.8	N/A	N/A	N/A	N/A	N/A	6.9 ± 1.4
C–reactive protein concentration (mg/L)	<4	18.61 ± 10.41 (7 patients, 77.77%) ↑	14.58 ± 22.17 (6 patients, 60%) ↑	32.28 ± 33.57 ↑	19.6↑	31.87 ± 11.71↑	2.97 ± 0.53↑	4.8 ± 4.8 (5 patients, 31.3%)↑	N/A	(10 patients) ↑	N/A	1.5	N/A	3.7 (1.9–7.8)
Albumin (ALB), g/L	40–55	N/A	N/A	31.26 ± 2.76↓	24.6↓	32.42 ± 1.53↓	N/A	N/A	N/A	N/A	N/A	N/A	N/A	N/A
ALT (U/L)	0–35	253.77 ± 690 (3 patients, 33.33%) ↑	9.52 ± 4.13	10.26 ± 4.48	N/A	(2 patients, 28.57%)↑	N/A	11.6 ± 5.0	N/A	N/A	N/A	N/A	N/A	N/A
AST (U/L)	0–35	171 ± 410.12 (3 patients, 33.33%) ↑	17.91 ± 5.73	20.22 ± 7.49	N/A	1 patient, 14.28%)↑	N/A	16.3 ± 5.2	N/A	N/A	N/A	N/A	N/A	N/A
ALP (U/L)	35–100	N/A	N/A	178.2 ± 69.05↑	N/A	N/A	N/A	N/A	N/A	N/A	N/A	N/A	N/A	N/A
Lactate dehydrogenase (U/L)	120–250	N/A	190.41 ± 31.49	263.8 ± 85.59 (1 patient, 20%)↑	544↑	279.4 ± 57.66 (5 patients, 71.42%)↑	N/A	N/A	N/A	N/A	N/A	N/A	N/A	N/A
D–dimer (ug/L)	<0.5	N/A	2.29 ± 1.85↑	1.36 ± 0.72↑	0.84↑	1.39 ± 0.49↑	4.35 ± 1.37↑	N/A	N/A	N/A	N/A	N/A	N/A	N/A
Confirmatory test done (SARS–CoV−2 quantitative RT–PCR)		Yes	Yes	Yes	Yes	Yes	Yes	Yes	Yes	Yes	Yes	Yes	Yes	Yes

* *Five patients who had lower lymphocyte count and two patients were normal but their lymphocyte values were not provided that information*.

**Figure 1:**
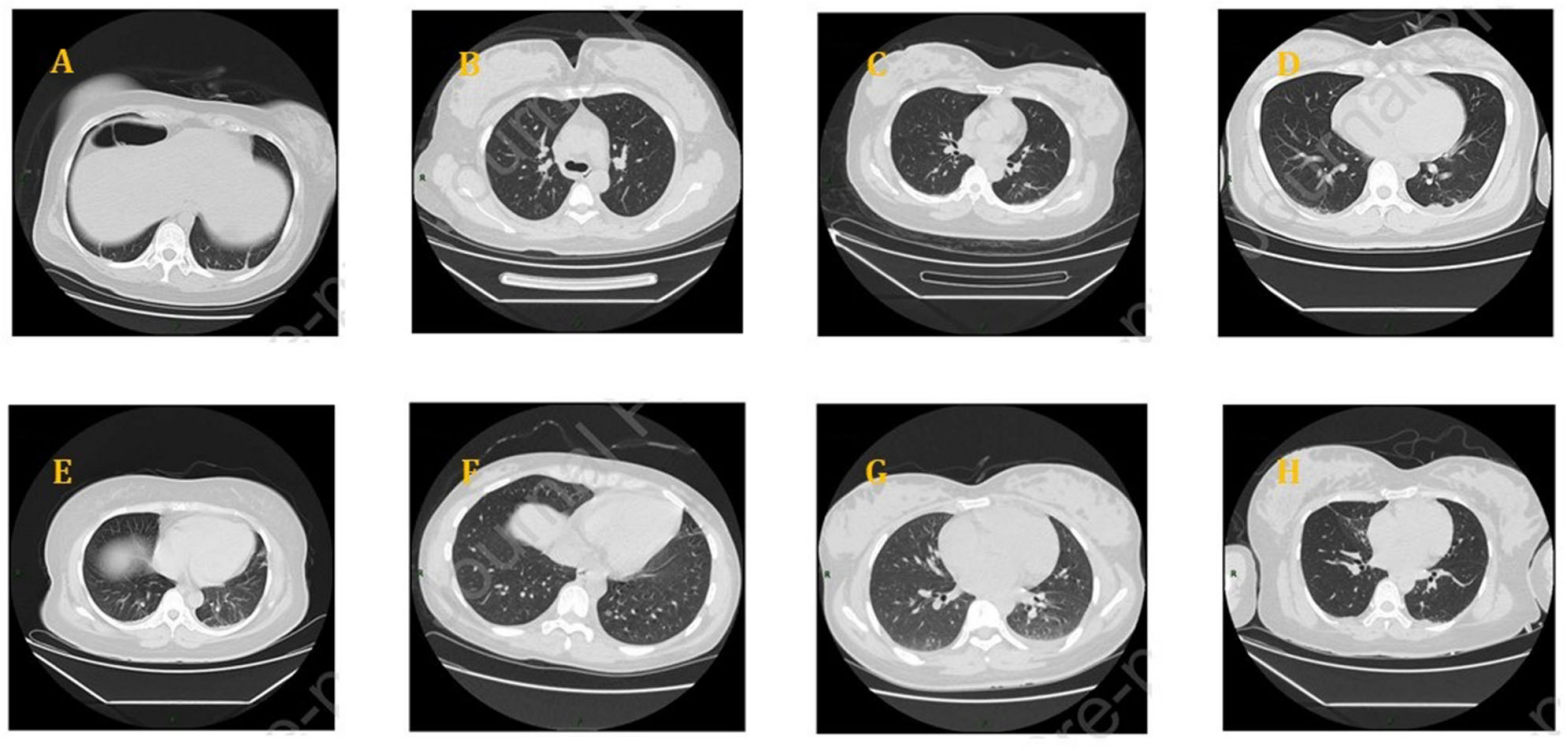
Chest CT scans of pregnant women with COVID-19: **(A)** bilateral lower lung cord, **(B)** a ground-glass nodule on the right upper lobe, **(C)** denser shadows scattered on the right lower lobe, **(D)** a little patchy shadow on the dorsal side of bilateral lower lungs and a little pleural effusion, **(E)** patchy shadows on bilateral lungs and a small amount of pleural effusion on both sides, **(F)** ground-glass density nodules on the left, **(G)** patches near the pleura, bilateral pleural effusion, and **(H)** ground glass and patch shadows scattered on the bilateral lungs.

All the studies mentioned that pregnant women underwent a pulmonary CT scan and showed abnormalities in different degrees including ground-glass opacity, patch-like shadows, fiber shadows, pleural effusion, and pleural thickening. There were no differences between the pregnant and non-pregnant groups. The involvement of lesions mostly was bilateral multi-lobe, and there was the peripheral, random, and diffuse distribution of pulmonary lesions (Figure 1). Each patient further went to a CT scan examination 3–4 days after their first examination during admission.

Our study shows no information about the fetus and neonatal death. Even neonatal asphyxia was not reported. Twenty-five newborns were pre-mature, and fifteen newborns reported a low birth weight. The range of birthweights was 1,520–4,050 g. However, the 1-min and 5-min Apgar scores were 7–10 and 8–10, respectively (Table 3). There were two newborns that showed turbidity of the amniotic fluid, and no baby died. Neonatal throat swab and breastmilk samples were examined for the presence of SARS-CoV-2 but all were negative.

**Table 3:** Neonatal outcomes of 235 COVID-19 patients.

	**Chen**	**Cao**	**Chen**	**Wang**	**Yu**	**Wu$**	**Li**	Chen^*^	**Liu**	**Zhu**	**Lee**	**Yang**	**Yang**
**Gestational age**													
Range (week, day)	36–39^+4^	33^+6^-40^+6^	38^+6^-40^+4^	30	37–41^+2^	8–40	38 ± 0.2	N/A	N/A	31–38^+4^	37^+6^	36–38^+2^	38.2 ± 2.3
**Birthweight (g)**													
Avg. ± SD	3,011.11 ± 621.53	3,072.5 ± 551.27	3,691 ± 295.47	1,830	3,264 ± 151.97	–	3,066.7 ± 560.2	–	N/A	2,423 ± 375.20	3,130	2,962.85 ± 660.80	3,063.2 ± 536.4
Range	1,880–3,730	2,050–3,800	3,235–4,050	–	3,000–3,500	–	N/A	–	N/A	1,520–3,800	–	1,880–3,800	N/A
Low birthweight (<2,500 g)	2	1	0	1	0	–	3	N/A	N/A	7	0	1	N/A
Pre-mature delivery	4	3	0	0	0	–	4	14/68	N/A	0	0	0	N/A
**Apgar score**													
**(1 min, 5 min)**													
Range	8–9 & 9–10	8–9 & 10	10 &10	–	8 & 10	–	–	N/A	8 & 9	7–10 & 8–10	9 & 10	8–9 & 9–10	N/A
Avg. ± SD	8.71 ± 0.48 & 9.71 ± 0.48	8.6 ± 0.53 & 10 ± 0	10 ± 0 & 10 ± 0	–	8 &10	–	9.6 ± 0.5 & 10.0 ± 0.0	N/A	8 & 9	8.6 ± 0.75 & 9.4 ± 0.89	9 & 10	8.28 ± 0.48 & 9.28 ± 0.48	N/A
Severe neonatal asphyxia	No	No	N/A	N/A	No	–	N/A	No	No	No	N/A	N/A	N/A
Amniotic fluid	N/A	Clear: 8, turbidity: 2	N/A	N/A	N/A	–	N/A	N/A	N/A	N/A	N/A	Negative: 5	N/A
Neonatal death	No	No	No	No	No	–	No	No	No	No	No	No	
Neonatal confirmatory test (COVID-19 qRT–PCR)	Negative	Negative	Negative	Negative	Negative	–	Negative	N/A	N/A	Negative	Negative	Negative	Negative

## Discussion

### Main Findings

This first comprehensive systematic review shows clinical, laboratory, radiological features, and neonatal outcomes from 235 pregnant women diagnosed with COVID-19. The clinical characteristics, laboratory, and radiological manifestations are similar in pregnant women with SARS-CoV-2 infection when compared with the non-pregnant or general population. In terms of clinical characteristics, the most common signs and symptoms of pregnant women were fever, fatigue, and cough. However, diarrhea, myalgia, and shortness of breath were also reported but to a lesser extent. Laboratory findings indicated that pregnant women with COVID-19 experienced a reduction of absolute lymphocyte counts and an increment of the CRP, erythrocyte sedimentation rate (ESR), and D-dimer; however, leucocytes were in normal range among those patients. When it comes to intrauterine vertical transmission from mother to children and neonatal deaths, no vertical transmission and neonatal death were reported. One hundred fifty six out of 235 pregnant had a cesarean (C-section). Severe pre-eclampsia, a history of C-section, placenta abruption, twins' pregnancy, and fetal distress were the main reasons for the C-section. SARS-CoV-2 infections were not indications for a C-section. However, panic regarding this novel disease might lead to choosing a C-section, but this was not reported or investigated.

### Public Health Implications

Coronaviruses (CoVs) are a single positive-stranded RNA virus that causes infection of humans (21). SARS-CoV-2 is one of the coronavirus species that emerged in China and which has rapidly spread throughout the world in 2020. The pandemic of SARS-CoV-2 infection has already created public health threats resulting in a significant proportion of deaths (22, 23). Pregnant women are more susceptible to respiratory pathogens and pneumonia and are at risk of adverse obstetrical outcomes like the pre-mature rupture of membranes (PROM), pre-term labor (PTL), and neonatal death. This is due to the immunosuppressive state as well as physiological differences (e.g., diaphragm elevation, increased oxygen consumption, and oedema of respiratory tract mucosa) which often leads to severe hypoxia (24, 25). Previous studies reported that the mortality rate for pregnant women with influenza (1918–1919 pandemic) was 37%; the rate of mortality was even higher (~50%) in patients who were in their 3rd-trimester of pregnancy (26). During the 1957–1958 Asian flu epidemic, pregnant women experienced a mortality rate of 10% which was 2-fold than that of non-pregnant women (27). Moreover, SARS and MERS were found to be associated with spontaneous miscarriages and maternal death (4, 28). Nearly 50% of pregnant women with SARS-CoV were admitted to the intensive care unit (ICU), and among them one-third required mechanical ventilation, and ~25% of patients died who admitted to ICU, or required mechanical ventilation (4). Approximately 57% of pregnant women with SARS-CoV who were in their first trimester experienced spontaneous miscarriages, and 80% of patients with late pregnancy had preterm deliveries. Approximately 80% of patients had an emergency cesarean due to inadequate maintenance of blood oxygen saturation, despite being on 100% oxygen (29). Limited data are available for pregnant women with MERS-CoV infection. Alfaraj et al. mentioned that the case fatality rate (CFR) of pregnant women with MERS-CoV was 27% but that was not significantly different when compared with the overall CFR of MERS-CoV in the general population (35%) (30).

Guan et al. (31) reported a 1.4% mortality rate of pregnant women with COVID-19. According to our study findings, pregnancy complications, fetal and neonatal outcomes of pregnant women with SARS-CoV-2 are much better than those of SARS-CoV and MERS-CoV. All the babies who tested for SARS-CoV-2 were negative; therefore, findings of our study do not support the possibility of vertical transmission of SARS-CoV-2 infection. Previous studies also showed that pregnant women with SARS infection had not experienced any vertical transmission from the mother to the newborn baby (4, 32). More attention must be paid to pregnant women because it has been reported that SARS-CoV-2 infection is linked to a cytokine storm (33) (elevated plasma concentrations of interleukins 2 (IL-2), IL-7, IL-10, interferon-γ-inducible protein 10 (IP-10, CXCL10), monocyte chemoattractant protein 1, macrophage inflammatory protein 1 alpha, and tumor necrosis factor α (TNF-α) that may lead to undesirable outcomes in pregnant women, especially in their first and third trimester as well as neonates. Maternal inflammation during pregnancy is also associated with fetal brain development and may lead to neuronal dysfunctions and behavioral phenotypes (34). Our study reported common signs and symptoms of pregnant women with COVID-19 which could increase attention-deficit/hyperactivity disorder in the newborn baby (35).

CT scan imaging is found to be an effective strategy to identify asymptomatic pregnant women with COVID-19 early as it shows the abnormality in patients (9). Timely identification and intervention of patients can reduce obstetrical complications such as miscarriage, intrauterine growth restriction, and pre-term delivery, and improve pregnancy outcomes. Symptomatic patients need to be admitted to a hospital quickly and isolated in the intensive care unit with a negative pressure room (36). In the process of fetal monitoring, regular fetal heart rate should be monitored electronically to assess the fetal status dependent upon the gestational age. Our study findings also show that there is no association between COVID-19 and pregnancy termination; therefore, a decision regarding the delivery process must be individualized. Patients with mild symptoms should be treated with the available treatment options and monitored until the condition has improved. However, patients with critical conditions or chance to intrauterine fetal demise or loss of both mother and baby should be warranted.

### Strengths

This current systematic review has several strengths. First, 13 studies with 235 patients COVID-19 were included in this study. The inclusion of a large number of pregnant women will help to get a more comprehensive understanding of COVID-19 in pregnancy. Second, COVID-19 may increase the risk of mother-to-child intrapartum transmission, but our findings so far show no possibility of intrapartum transmission. Third, this study presents clinical characteristics and maternal and fetal outcomes of COVID-19 in late pregnancy from different country's perspectives.

### Comparison With Recent Findings

The findings and knowledge gained from our study suggests that pregnant women are particularly susceptible to poor outcomes and there was no evidence of vertical transmission and C-section. Since, several articles have reported that the possibilities of vertical transmission (37, 38), highlighting the importance of long-follow up of newborns (39). To clarify the vertical transmission and neonatal outcomes due to SARS-CoV-2 infection; we further compared our findings with other recently published systematic review and meta-analyses (38, 40–50). Raschetti et al. (38) evaluated neonatal outcomes from 74 published articles in where 30% of neonates were reported in the possible vertical transmission and 70% were from environmental sources (Table 4). It is just possible that we cannot eliminate but controversy still is going on whether SARS-CoV-2 infection transmits from the mother to the fetus. However, the most common complications of pregnant women were pre-eclampsia, fetal distress, and PROM. Majority of pregnant women went for C-section but there is no relation between COVID-19 infections or vertical transmission and C-section (Table 5). Chest computed tomography (CT) is the imaging is now being considered as primary option for diagnosing COVID-19 in pregnant women because it help to diagnosis disease in an early stage (52). Chest computed tomography (CT) is considered as gold standard and the performance is higher than that of the reverse transcription polymerase chain reaction for disease diagnosis (53). Lung ultrasound (LUS), a radiation-free point-of-care diagnostic tool, has now been suggested as an accurate tool to detect COVID-19 because LUS is more sensitive to detect COVID-19 than that of chest X-ray (54). Our study is different from other studies because we have reported comprehensive clinical features, laboratory findings, and neonatal outcomes. Our study findings and findings from recently published systematic reviews may assist clinicians to understand the nature of the disease, and to make appropriate decisions when treating pregnant women with COVID-19.

**Table 4:** Basic characteristics, findings and suggestions from recent literatures.

**Author**	**Study type**	**No. of included studies**	**Number of pregnant women and neonates**	**Findings**	**Suggestions**
Raschetti et al. (38)	Systematic review	74	PW = N/A; *N* = 176	Infections mainly occur postnatally through environmental exposure. However, vertical transmission might be occurred in ~30% of neonates.	Following hygiene advice and proper use of personal protective equipment is important to reduce the risk of transmission.
Akhtar et al. (40)	Systematic review	22	PW = 156; *N* = 108	Increased risk in pregnancy complications such as pre-term birth, PPROM, and may possibly lead to maternal death in rare cases. No evidence of vertical transmission.	Appropriate caution should be taken and further investigate and monitor possible infection in the neonates born to COVID-19–infected mothers.
Chamseddine et al. (42)	Systematic review	48	PW = 248, *N* = 201	There are no evidence of vertical transmission; although, some neonates were tested positive. It can be happened in the hospital or at home environment after birth.	Strictly follow guidelines.
Gao et al. (43)	Systematic review and meta–analysis	24	PW = 236	No evidence that COVID-19 can spread through vertical transmission.	Vertical transmission is possible in patients with their first or second trimester or where long–delivery interval. Therefore, pregnant women remain alert for the possibility of vertical transmission.
Gatta et al. (44)	Systematic review	6	PW = 51; *N* = 48	COVID-19 was associated with increased risk of respiratory insufficiency in late pregnancies. However, there is no strong evidence for vertical transmission.	Future studies are warranted to provide a detail information on maternal and fetal conditions, as well as the rationale for obstetrical interventions.
Khalil et al. (45)	Systematic review and meta–analysis	86	PW = 2,567	COVID-19 was associated with an increased risk of iatrogenic pre-term birth and cesarean delivery. Vertical transmission probably occurs in neonates.	Comprehensive assessment is required to support or refute the risk of vertical transmission.
Smith et al. (46)	Systematic review	9	PW = 92; *N* = 37	COVID-19 was associated with increased risk of pre-term births, low birth weight, C-section. The evidence of vertical transmission is vague.	A multidisciplinary approach is recommended to monitor patients.
Lopes de Sousa et al. (47)	Systematic review	49	PW = 755; *N* = 598	SARS–CoV−2 may be associated with several pregnancy complications. However, there is no potential evidence of vertical transmission	Recommend a comprehensive monitoring and test pregnant women before deliver or first contact with newborn.
Di Toro et al. (51)	Systematic review and meta–analysis	24	PW = 1,104, *N* = 444	COVID-19 did not significantly influence the Pregnancy. Although, higher rate of C-section had been reported but there was no link to their association. Furthermore, no clear evidence of vertical transmission.	Pregnant women with COVID-19 should not be taken to C-section irrationally. More studies are needed to confirm possible risk of pregnant women with SARS–CoV−2 infection.
Trocado et al. (48)	Systematic review	8	PW = 95; *N* = 51	Pregnant women were more susceptible to SARS–CoV−2 infection and faced several complication. Moreover, vertical transmission to neonate cannot be ignored.	More researches are warranted to get clear information about the impact of COVID−19 in pregnant women.
Turan et al. (49)	Systematic review	63	PW = 637; *N* = 318	COVID-19 was associated with poor maternal and neonatal outcomes. Vertical transmission can be occurred but there is no clear evidence.	Counseling is needed to the pregnant women with COVID−19.
Yee et al. (50)	Systematic review and meta-analysis	11	PW *=* 9,032; *N* = 338	SARS-CoV-2 infection may increase several complications among pregnant women including neonatal death. The possibility of vertical transmission to neonates can be ruled out.	More studies are needed to confirm or refute some potential harms for pregnant women.
Zaigham and Andersson (41)	Systematic review	18	PW = 108	COVID-19 was associated with an increased rate of poor neonatal outcomes. The possibility of vertical transmission cannot be ignored.	Careful monitoring and measurements are needed to reduce the fatal rate.

*PW, Pregnant women; N, Neonate*.

**Table 5:** Pregnant women and neonatal outcomes compression among recent literatures.

**Author**	**Pregnant women**												**Neonatal outcome**			
	**Complications**										**Delivery methods**		**Characteristics**			
	**Gestational hypertension**	**Gestational diabetics**	**Pre-eclampsia**	**Fetal distress**	**PROM**	**Pre-mature**	**Uterine scarring**	**Placental abruption**	**Hypothyroidism**	**Anemia**	**C-section**	**Vaginal birth**	**Birthweight (g)**	**Pre-mature delivery**	**COVID-19 tested postitive**	**Neonatal death**
Raschetti et al. (38)	N/A	N/A	N/A	N/A	N/A	Yes	N/A	N/A	N/A	N/A	66 (37.5%)	N/A	2,782 (900–4,500)	Yes	Yes (~30 percent)	Yes (3 out of 176)
Akhtar et al. (40)	N/A	N/A	N/A	14%	8%	Yes	N/A	N/A	N/A	N/A	66 (42.30%)	19 (12.17%)	N/A	Yes	N/A	Yes (10 out of 108
Chamseddine et al. (42)	N/A	6.9%	6.1%	35.3%	N/A	N/A	N/A	6.5%	N/A	N/A	201 (89%)	N/A	N/A	Yes	Yes	2.5%
Gao et al. (43)	N/A	N/A	N/A	29%	N/A	N/A	N/A	N/A	N/A	N/A	65%	N/A	N/A	N/A	No	N/A
Della Gatta et al. (44)	Yes	N/A	Yes	N/A	26%	Yes	N/A	N/A	N/A	N/A	Yes	N/A	N/A	Yes	No	N/A
Khalil et al. (45)	N/A	N/A	N/A	6.30%	N/A	Yes	N/A	N/A	N/A	N/A	390 (52.31%)	N/A	N/A	Yes	Yes (2.5%)	Yes (4 out of 688)
Smith et al. (46)	N/A	N/A	N/A	61.11%	N/A	63.83%	N/A	N/A	N/A	N/A	40 (80%)	3 (6%)	(21) 2,743.81 (± 676.34)	N/A	Yes (4.76%)	N/A
Lopes de Sousa et al. (47)	N/A	N/A	N/A	N/A	N/A	20%	N/A	N/A	N/A	N/A	379 (65%)	N/A	N/A	Yes	Yes (2%)	Yes (10 out of 598)
Di Toro et al. (51)	N/A	N/A	7%	N/A	10%	23%	N/A	N/A	N/A	N/A	713 (85%)	686 (14%)	N/A	N/A	Yes (2%)	N/A
Trocado et al. (47)	Yes	Yes	1%	N/A	N/A	N/A	N/A	N/A	N/A	N/A	47 (94%)	3 (6%)	2,292.53 g	N/A	Yes (2%)	Yes (1 out of 48)
Turan et al. (49)	Yes	Yes	N/A	46.7%	N/A	Yes	N/A	N/A	N/A	N/A	403 (84.1%)	76 (15.9%)	N/A	Yes	Yes (2%)	Yes (5 out of 479)
Yee et al. (50)	Yes	Yes	N/A	15.1%	2.4%	28.6%	N/A	M/A	N/A	N/A	N/A	N/A	2,855.9	Yes	Yes (2%)	Yes (5 out of 154)
Zaigham and Andersson (41)	N/A	N/A	N/A	N/A	N/A	N/A	N/A	N/A	N/A	N/A	86 (92%)	7(8%)	N/A	N/A	Yes (1%)	Yes (1 out of 87)

### Conclusion

This study shows that the clinical characteristics of pregnant women with COVID-19 were varied, with the main signs and symptoms being fever, cough, and sore throat. Conducting a CT scan would be helpful to classify as early or progressive stages, especially in asymptomatic patients, and can thus reduce transmission. As pregnant women are susceptible to COVID-19, lymphocyte count, CRP, albumin, and D-diameter could thus be primary indicators for pregnant women with COVID-19. Recent evidence suggests that there is a possibility of vertically transmission from infected pregnant mothers to their fetuses. C-section should not be routinely recommended anymore according to latest evidences and, in any case, decisions should be taken after proper discussion with the family. Further studies are needed to confirm long-term outcomes and potential mother-to-child vertical transmission with higher sample sizes.

## Data Availability Statement

The raw data supporting the conclusions of this article will be made available by the authors, without undue reservation.

## Author Contributions

MI, TP, HY, C-WW, and W-SH: data collection and analysis. MI: writing. SA, HS, BA, ML, WJ, and BW: revising. Y-CL: supervision and funding. All authors contributed to the article and approved the submitted version.

## Conflict of Interest

The authors declare that the research was conducted in the absence of any commercial or financial relationships that could be construed as a potential conflict of interest.
