# Supplementary material for: Clinical Characteristics and Neonatal Outcomes of Pregnant Patients With COVID-19: A Systematic Review
Source: Front Med (Lausanne). 2020 Dec 3;7:573468. doi: 10.3389/fmed.2020.573468 (PMC7772992; doi:10.3389/fmed.2020.573468)
Supplement: Supplementary file 1 [file Data_Sheet_1.docx]

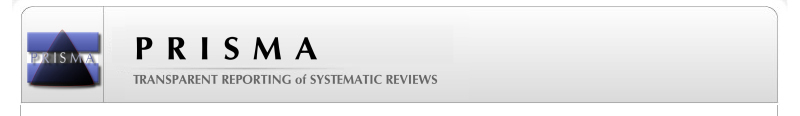
**PRISMA 2009 Flow Diagram**

Records identified through EMBASE, PubMed, Google, Google scholar, Scopus
(n = 237 )

Additional records identified through other sources
(n = 0)

Records screened
(n = 135)

Records after duplicates removed
(n = 135 )

## Identification

## Eligibility

## Included

## Screening

Records excluded after evaluation of titles and abstract
(n = 88 )

Full-text articles excluded (n=34):

Did not meet inclusion criteria = 23

Did not provide appropriate characteristics of pregnant women = 7

Review= 3

Duplication=1

Full-text articles assessed for eligibility
(n = 47 )

Studies included in qualitative synthesis
(n = 13 )

Studies included in quantitative synthesis
(n = 0 )

**Figure 1:** PRISMA flow diagram for study selection
